# Targeting acetylcholine: a novel strategy for treating lung adenocarcinoma

**DOI:** 10.1186/s12920-026-02412-8

**Published:** 2026-07-03

**Authors:** Suoni Li, Yuxin Song, Hui Wang, Jiequn Ma, Jie Bai, Xinming Xie, Bo Guo, Zhao Wei, Yu Yao

**Affiliations:** 1 Department of Oncology, The First Affiliated Hospital of Xi’an Jiaotong University, Xi’an, 710061 China; 2 Department of Oncology, Shaanxi Provincial Tumor hospital, Xi’an, Shaanxi 710061 China; 3 Department of Cell Biology and Genetics, School of Basic Medical Sciences, Xi’an Jiaotong University Health Science Center, Xi’an, China; 4 Institute of Genetics and Developmental Biology, Translational Medicine Institute, School of Basic Medical Sciences, Xi’an Jiaotong University Health Science Center, Xi’an, China; 5 School of Life Science and Technology, Xi’an Jiaotong University Health Science Center, Xi’an, China; 6 Department of Medicinal Chemistry, School of Pharmacy, Air Force Medical University, Xi’an, 710032 China; 7 Department of Respiratory and Critical Care Medicine, the First Affiliated Hospital of Xi’an Jiaotong University, Xi’an, 710061 PR China

**Keywords:** Acetylcholinesterase, Lung adenocarcinoma, Acetylcholinesterase inhibitors, Compound synthesis, Cell function

## Abstract

**Background:**

Lung cancer remains one of the deadliest cancers, both in terms of the incidence and mortality rates. Although a combination therapy comprising immune checkpoint inhibitors and chemotherapy has become the standard therapy for driver gene-negative lung adenocarcinoma, its efficacy is yet to be further improved. Additionally, new treatment methods still need to be developed. Acetylcholinesterase (AChE) has emerged as a potential therapeutic target in various cancers. However, its role in lung adenocarcinoma remains poorly understood.

**Objective:**

This study aims to investigate the role of AChE in the progression of lung adenocarcinoma and to design and synthesize small-molecule compounds targeting AChE for exploring their potential as novel therapeutic agents.

**Methods:**

AChE is significantly overexpressed in lung adenocarcinoma. Therefore, we synthesized two novel AChE inhibitors and characterized them by nuclear magnetic resonance and high-resolution mass spectrometry. Subsequently, two inhibitors were added to lung adenocarcinoma A549 and H1975 cells to detect changes in their biological behaviors such as cell proliferation, apoptosis, cell cycle, and colony-formation ability. Simultaneously, a mouse transplant tumor model was constructed and an AChE inhibitor was injected intraperitoneally to observe changes in the volume and weight of the mouse transplant tumor.

**Result:**

Our AChE inhibitors showed significant cytotoxicity against A549 and H1975 cells. They can effectively inhibit cell proliferation, induce apoptosis, prevent cell cycle progression, and reduce colony-formation ability. The mouse transplant tumor model confirmed that they can inhibit cell proliferation. The tumor volume and weight were significantly reduced in the intraperitoneal injection inhibitor group. Notably, the inhibitor did not cause pathological damage to normal organs.

**Conclusion:**

Our novel AChE inhibitors can potentially be used to treat lung adenocarcinoma and to develop a promising new direction for future lung adenocarcinoma treatments.

**Supplementary Information:**

The online version contains supplementary material available at https://doi.org/10.1186/s12920-026-02412-8.

## Introduction

Lung cancer is one of the most malignant tumors with very high incidence and mortality rates worldwide. Non-small cell lung cancer (NSCLC) accounts for 80–85% of all types of lung cancer [1]. Lung adenocarcinoma is a common histological subtype of NSCLC. In the past decade, advances in the treatment of driver gene-positive advanced lung adenocarcinoma, including the use of epidermal growth factor receptor (EGFR) [2] and anaplastic lymphoma kinase (ALK) [3], have significantly improved the survival rate of patients with driver gene-positive lung adenocarcinoma. However, effective therapeutic strategies for driver gene-negative advanced lung adenocarcinoma have still not been developed.

Immunotherapy has proven to be a promising treatment for NSCLC in recent years, especially in patients with driver gene-negative advanced NSCLC. However, the efficacy of immune checkpoint inhibitors is still limited. For example, the objective response rate (ORR) of immune monotherapy in patients with advanced NSCLC with PD-L1 ≥ 50% is only 44% [4], while it is < 30% in patients with a tumor mutation burden cutoff of ≥ 10/Mb [5]. Although immune combination chemotherapy significantly improved the ORR (50–70%) [6], high-dose chemotherapy reduces the number and damages the function of immune cells, especially those of CD8 + T cells, resulting in a shorter duration of efficacy (DoR) compared to that achieved with immunotherapy alone [7]. In addition, some patients are not suitable candidates for immune checkpoint inhibitor treatment. Therefore, other treatment methods should be explored.

Acetylcholinesterase (AChE), a member of the alpha/beta hydrolase fold superfamily of proteins, is a serine hydrolase responsible for terminating transmission at cholinergic synapses by rapidly hydrolyzing the neurotransmitter acetylcholine (ACh) [8]. The gene for encoding AChE is located at 7q22 in the human genome [9]. Although AChE is an essential enzyme primarily expressed in the central and peripheral nervous systems, different isoforms of AChE are also found in various cell types. In mammals, three different subtypes of AChE mRNA are formed through alternative splicing [10]. Tail type AChE isoforms (AChE-T) are mainly expressed in brain and muscle tissues [11]. The dimer H subtype (AChE-H) is common in human red blood cells [12]. Reading type AChE (AChE-R) can generate hydrophilic AChE monomers at cholinergic synapses to cope with stress [13].

An increasing number of studies have found that AChE, a key regulatory factor in cholinergic neurotransmission, may play a crucial role in cancer by regulating cholinergic neurotransmission. Aberrant AChE expression, gene amplification, and mutations have been reported in several tumor types, including liver cancer, colorectal cancer, breast cancer, and lung cancer [14]. The rationale for investigating AChE in lung adenocarcinoma rests on two critical disease phenotypes: uncontrolled proliferation and apoptosis resistance, both of which are profoundly influenced by autocrine/paracrine ACh signaling. In many epithelial cancers, ACh acts as a growth factor, stimulating tumor cell proliferation via nicotinic and muscarinic receptors, while AChE terminates this signaling. In lung adenocarcinoma, our preliminary database analysis (see Results) and independent proteomic studies [15] consistently reveal AChE overexpression, suggesting a compensatory or dysregulated cholinergic loop that fuels tumor progression. Disrupting this loop with AChE inhibitors may therefore simultaneously reduce proliferation, induce apoptosis, and impair clonogenic survival—the very phenotypes assessed in this study. Wang et al. demonstrated decreased expression of AChE in liver cancer and developed a transferrin-modified liposome delivery system to target AChE expression, which afforded promising therapeutic effects [16]. Conversely, in lung adenocarcinoma, the cholinergic system appears hyperactive. Recently, Li et al. showed that AChE facilitates brain metastasis through immune suppression and blood–brain barrier disruption, while choline acetyltransferase enhances the invasiveness of cancer cells [17]. These findings position AChE not only as a tumor promoter but also as a druggable vulnerability. Unlike prior studies that mainly examined AChE expression or used existing inhibitors (e.g., donepezil and galantamine), often repurposed from neurology, our work introduces two chemically novel, purpose-designed small-molecule AChE inhibitors, L1R1-6 and L3R1-3, and provides a comprehensive functional validation both in vitro and in a xenograft model. This development represents a significant addition to the field by offering new chemical entities with translational potential specifically tailored for lung adenocarcinoma. Now the question arises whether inhibiting the expression of AChE in lung adenocarcinoma is of any therapeutic value.

This study analyzes AChE expression in the TIMER2.0 database. It describes the synthesis of novel AChE inhibitors to evaluate their therapeutic potential in lung adenocarcinoma cell lines and in a transplanted tumor model of lung adenocarcinoma in mice. Our study aims to elucidate the functional role of AChE in lung adenocarcinoma and assess its viability as a molecular target for innovative treatment strategies.

## Methods

### Gene expression analysis

Gene expression data were obtained from the TIMER2.0 (http://timer.cistrome.org/) database to evaluate the mRNA levels of AChE in different types of cancers and in normal tissues.

### Cell culture

A549 and H1975 lung adenocarcinoma cells were cultured in the DMEM (Gibco BRL, NY, USA) supplemented with 10% fetal bovine serum (FBS, Dining, Beijing, China) and 1% penicillin–streptomycin at 37 °C in a 5% CO₂ incubator. The cells were subcultured every 2–3 days using 0.25% trypsin–EDTA. The human lung adenocarcinoma cell lines A549 and H1975 were provided by the Biomedical Experiment Center of Xi’an Jiaotong University.

### Synthesis and characterization of AChE inhibitors

First, paracetamol was modified by introducing an ethylamino side chain via amidation, resulting in the formation of intermediate product 3a. Next, the hydrolysis or reduction deprotection of amide bond (-NHCOCH_3_) of 3a was carried out, which generated the key intermediate R1-3. At this time, the substituents on the benzene ring were phenolic hydroxyl (-OH), free amino group (-NH_2_), and ethylamino group (-N(C_2_H_5_)_2_), all of which maintained a 1,2,4-trisubstituted structure. Finally, nucleophilic substitution condensation was carried out, which afforded a quinoline oxazine ring system (L3R1-3). This step involved intermolecular reactions between R1-3 (containing free amino groups) and L3 (chlorinated quinolone structure). The free amino group (-NH_2_) of R1-3 behaved as a nucleophile to attack the chlorine (-Cl^−^) adjacent to the carbon atom in the quinoline ring of L3, resulting in the final product L3R1-3. L1R1-6, an analogous compound, was synthesized by replacing L3 with L1 and replacing R1-3 with R1-6. Structural validation was performed using nuclear magnetic resonance (NMR) and high-resolution mass spectrometry (HRMS).

### Molecular docking simulations

The Schrödinger 2024’s Glide module was used for molecular docking to clarify how high-activity drugs bond to AChE. The synthesized molecules L1R1-6 and L3R1-3 were extensively assessed for their possible binding postures and docking scores.

### Cell viability assays

#### CCK-8 assay

Cells were seeded in 96-well plates and treated with varying concentrations of L1R1-6 and L3R1-3. After 24 h, 10 µL of CCK-8 solution (Fudebio-tech, Hangzhou, China) was added per well. Absorbance was measured at 450 nm using a microplate reader (BMG Labtech, Germany). The IC₅₀ values were calculated from the dose–response curves.

#### Live cell workstation

A549 and H1975 cells were seeded at 2 × 10³ cells/well in 96-well plates. Cell proliferation was monitored in real-time using the Cytation™ 5 Cell Imaging Multi-Mode Reader (BioTek, VT, USA) and analyzed with Gen5 Image software (v3.08).

### Cell apoptosis assay

Apoptotic cells were detected through Annexin V-FITC/PI staining (Beyotime, Shanghai, China). The cells were harvested, stained according to the manufacturer’s protocol, and analyzed within 1 h by flow cytometry (NovoCyte, ACEA Biosciences, USA).

### Cell cycle assay

Post-treatment, cells were fixed in 75% ethanol for 24 h at 4 °C. After RNase (MP Biomedicals, CA, USA) and PI (MedChemExpress, NJ, USA) staining, cell cycle distribution was analyzed by flow cytometry.

### Colony-formation assay

Following treatment, 1,000 cells were plated into 12-well plates and incubated for 1–2 weeks. The resulting colonies were fixed with 4% paraformaldehyde, stained with 0.1% crystal violet, and quantified using absorbance measurements at 570 nm after dissolving in 33% glacial acetic acid.

### Constructing a tumor xenograft model and intraperitoneal injection of drug L3R1-3 in mice

#### Tumor xenograft models

To evaluate the inhibitory effects of L3R1-3 on A549 cells, we developed a xenograft model using BALB/c nude mice (4-week-old), which were obtained from GemPharmatech Co., Ltd. (Nanjing, Jiangsu Province, China). We injected 1.5 × 10^7^ of A549 cells subcutaneously into each dorsal flank of mice (*n* = 15). The animals were housed in a 60% humidified atmosphere at 24 ℃, under a 12-h light/dark cycle with free access to purified drinking water and food.

#### Intraperitoneal injection of drug L3R1-3 in mice

After successful construction of the transplanted tumor model, the nude mice were divided into three groups: MOCK group, L3R1-3 25 mg/kg group, and L3R1-3 50 mg/kg group. Next, 150 µL of 0.5% acetic acid solution, 25 mg/kg of L3R1-3 solution, and 50 mg/kg of L3R1-3 solution were injected into the abdominal cavity of the aforementioned three groups of nude mice, once every two days. On the 14th, 16th, 18th, 20th, 22nd, 24th, 26th, and 28th days after vaccination, the length and width of the tumors in these three groups were measured using a Vernier caliper. After 28 days, the BALB/c nude mice were anesthetized by intraperitoneal injection of pentobarbital at a concentration of 1.5 mg/mL. They were then euthanized via cervical dislocation, and the tumor was dissected and weighed. The measurement data were collected, the tumor volume was calculated (0.5 × tumor length × tumor width × tumor width), and the tumor growth curve was plotted.

#### Histological examination of organ tissues in nude mice stained with hematoxylin and eosin (H&E)

The heart, liver, spleen, lungs, and kidneys of all nude mice with transplanted tumors were dissected to evaluate whether L3R1-3 molecules have pathological effects on important organs using H&E staining. The tissues were rinsed free of blood before fixation and rapidly preserved in a 4% paraformaldehyde solution at room temperature for 12 h. A formalin-fixed tissue sample was embedded in paraffin, which was then sectioned into 4-mm sections. These sections were subsequently incubated at 60 °C for 2 h, followed by de-paraffinization using xylene. Afterward, the sections were rehydrated using an alcohol series. Subsequently, hematoxylin staining (5–10 min) was performed to stain the nuclei, which was then differentiated using a short wash of hydrochloric acid ethanol (several seconds) and finally blued using alkaline solution. This step was followed by the staining of the cytoplasm with eosin (1–3 min). Finally, the slices were sealed and stored. After gradient dehydration and transparency, the slices were sealed with neutral gum.

### Statistical analysis

Data were analyzed using GraphPad Prism 8.0. Statistical significance between groups was determined using one-way ANOVA or *t*-test, and *p*-values < 0.05 were considered statistically significant.

## Results

### AChE is overexpressed in lung adenocarcinoma

**Fig. 1:**
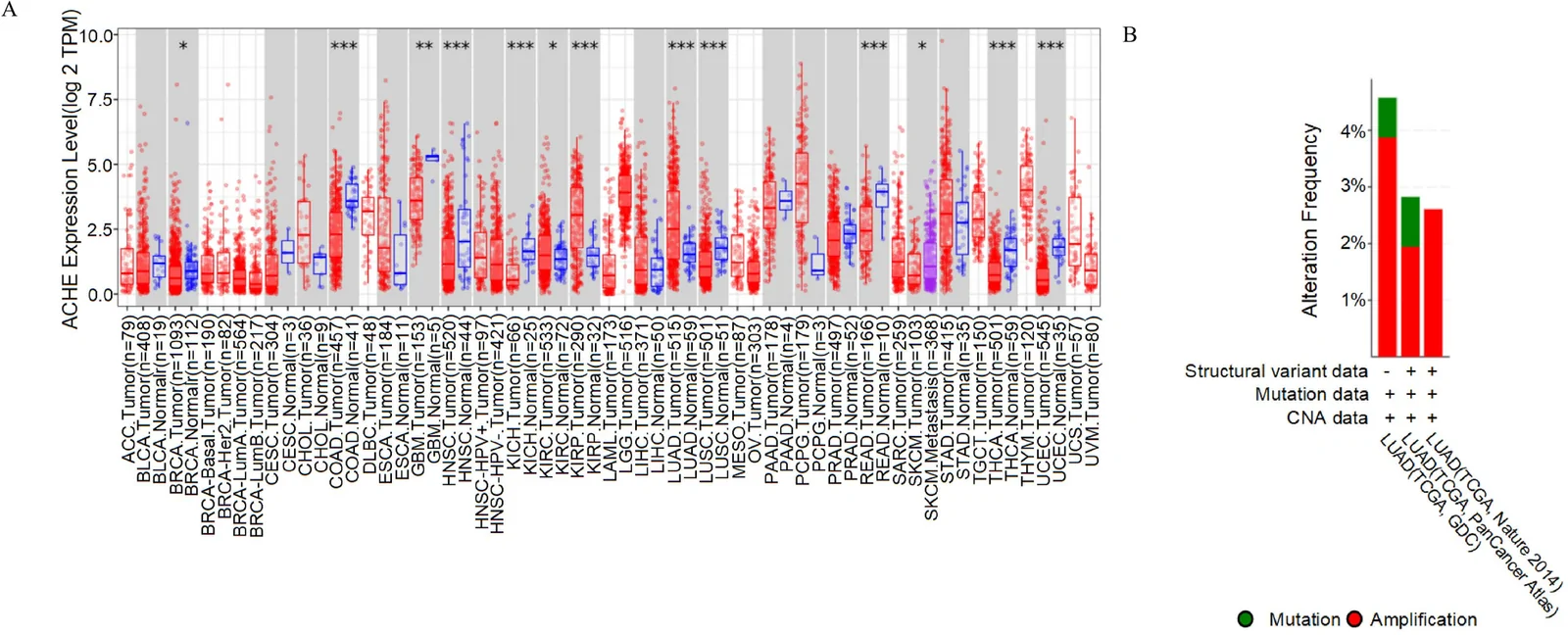
Expression landscape of acetylcholinesterase (AChE) in lung adenocarcinoma. **A** TIMER2.0 database analysis of AChE mRNA expression in tumor tissues. **B** Expression of AChE in lung adenocarcinoma

An analysis of the TIMER 2.0 database revealed that the AChE expression was significantly higher in lung adenocarcinoma tissues compared to that in normal tissues (*p* < 0.05; Fig. 1A and B).

### Synthesis and characterization of AChE inhibitors

**Fig. 2:**
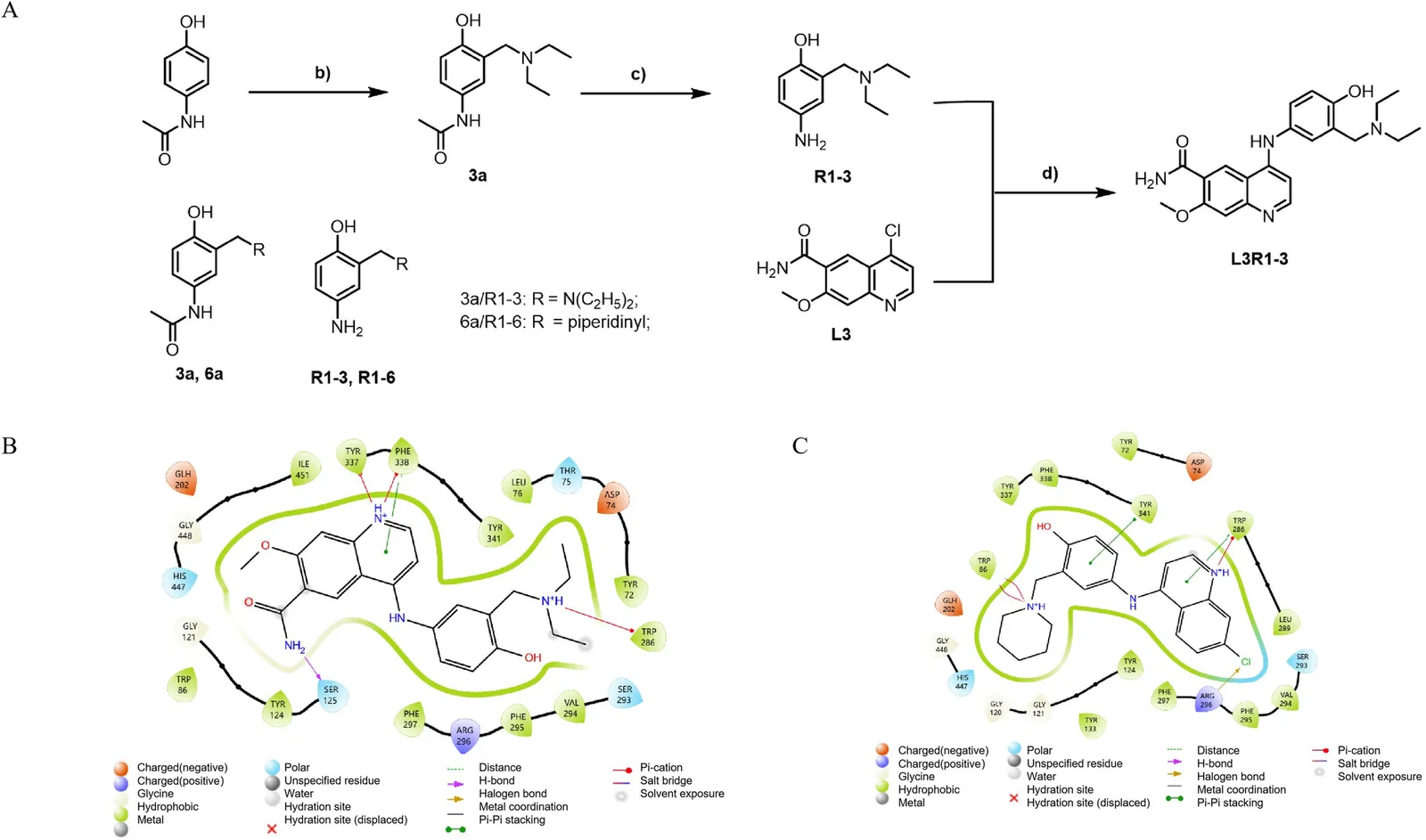
Synthesis and validation of acetylcholinesterase (AChE) inhibitors. **A** Synthetic scheme for the preparation of L3R1-3 and L1R1-6. **B** Molecular docking visualization of L3R1-3 binding to AChE. **C** Molecular docking visualization of L1R1-6 binding to AChE

**Fig. 3:**
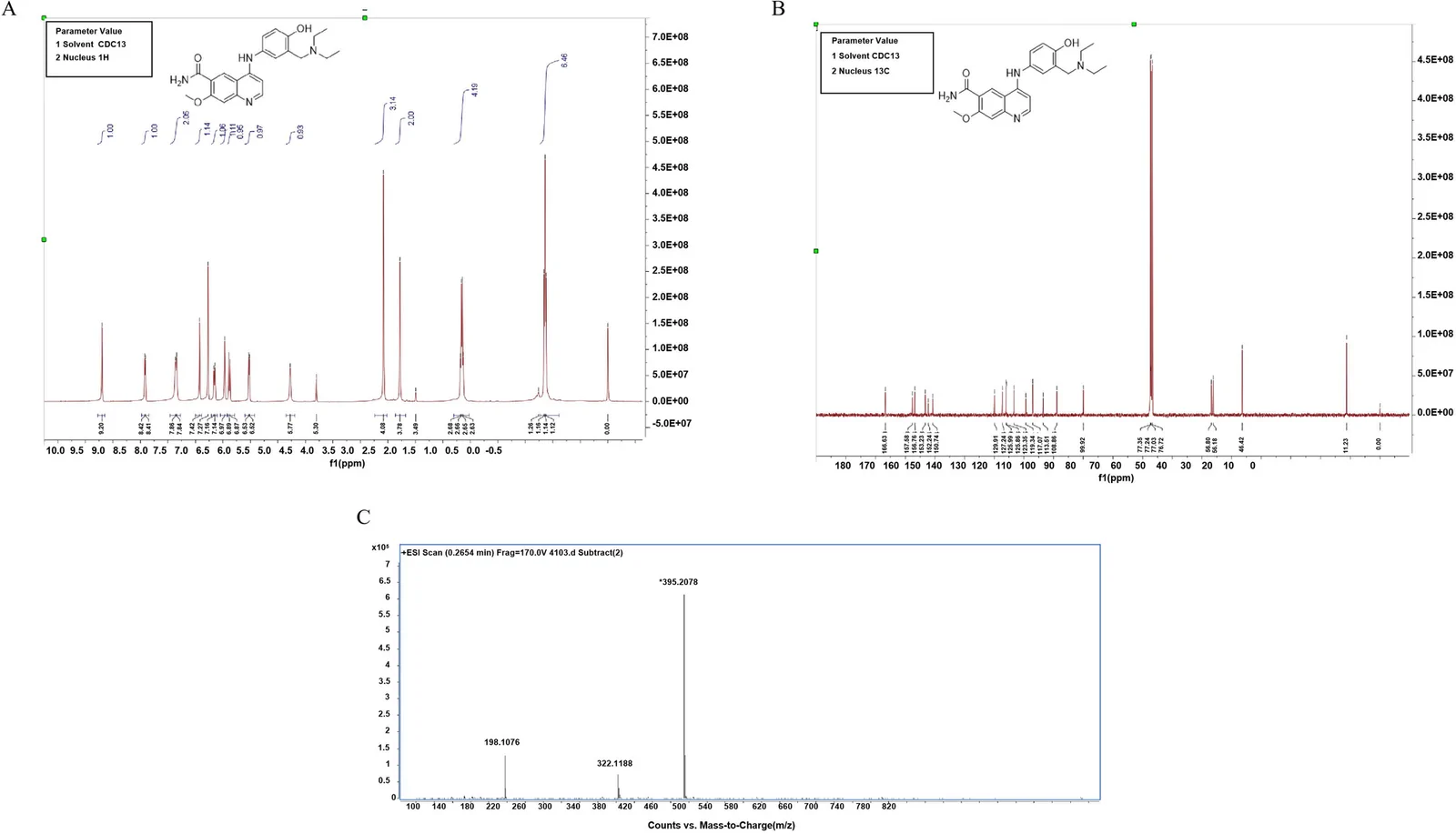
Spectral characterization of L3R1-3. **A** 1H NMR spectrum. **B** 13C NMR spectrum. **C** HRMS (ESI+) spectrum

**Fig. 4:**
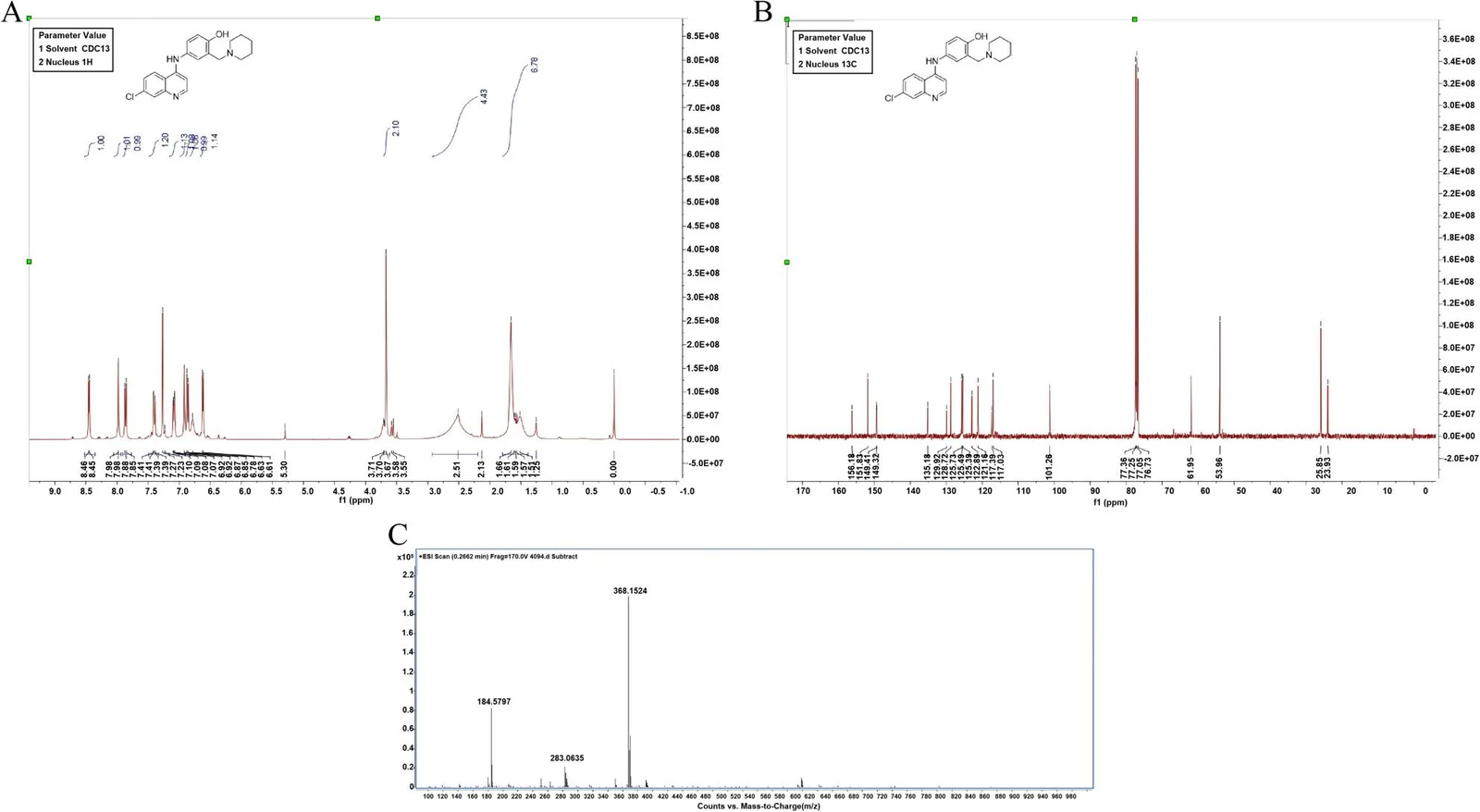
Spectral characterization of L1R1-6. **A** 1H NMR spectrum. **B** 13C NMR spectrum. **C** HRMS (ESI+) spectrum

We synthesized two novel AChE inhibitors—L1R1-6 and L3R1-3—to study the higher overexpression of AChE in lung adenocarcinoma (Fig. 2A). Molecular docking demonstrated favorable binding affinities of L1R1-6 and L3R1-3 to AChE, supporting their potential as AChE-targeting agents (Fig. 2B, C). Structural confirmation was performed using NMR and HRMS (Figs. 3 and 4).

### Determination of IC_50_ values for AChE inhibitors

**Fig. 5:**
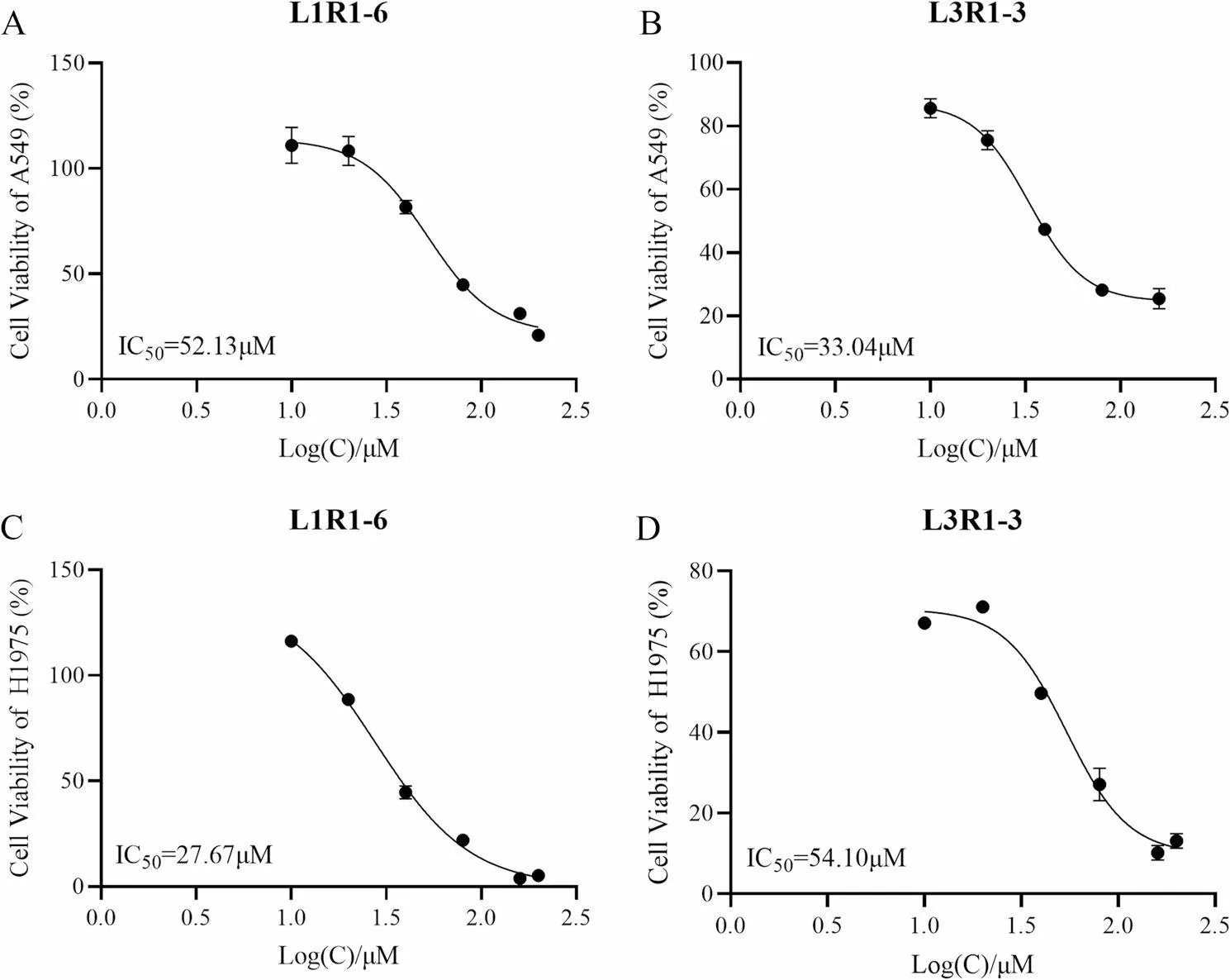
Determination of IC50 values for acetylcholinesterase (AChE) inhibitors using the CCK-8 assay

Treatment of A549 cells with L1R1-6 and L3R1-3 afforded IC₅₀ values of 52.13 and 33.04 µM, respectively (Fig. 5A and B), while that of H1975 cells yielded IC₅₀ values of 27.67 and 54.10 µM, respectively (Fig. 5C and D). Based on these IC₅₀ results, we selected L3R1-3 to treat A549 cells and L1R1-6 to treat H1975 cells in the subsequent experiments.

### AChE inhibitors suppress the growth of lung adenocarcinoma cells

**Fig. 6:**
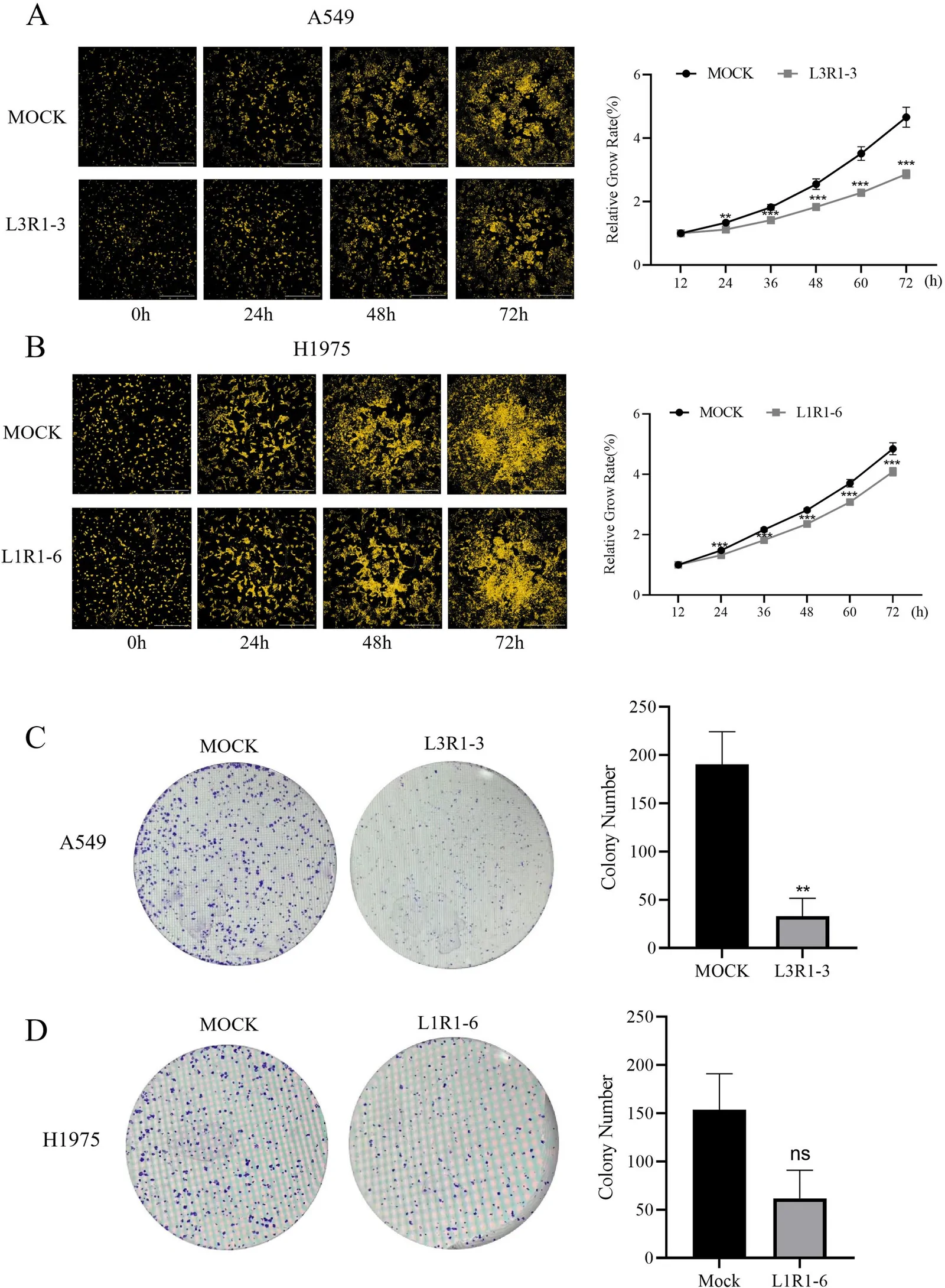
Functional effects of acetylcholinesterase (AChE) inhibitors on lung adenocarcinoma cells. **A** Monitoring the effect of different concentrations of L3R1-3 on the proliferation of A549 cells. **B** Monitoring the effect of different concentrations of L1R1-6 on the proliferation of H1975 cells. **C** Detection of the effect of L3R1-3 treatment on A549 cell colony formation. **D** Detection of the effect of L1R1-6 treatment on H1975 cell colony formation

At IC_50_ concentrations, both L1R1-6 and L3R1-3 showed lower cytotoxicity to normal cells than they did to cancer cells in both A549 and H1975, indicating their potential selectivity. L3R1-3 significantly inhibited cell proliferation in A549 (*p* < 0.05; Fig. 6A), while L1R1-6 significantly inhibited cell proliferation in H1975 (*p* < 0.05; Fig. 6B). Colony-formation assays showed that L3R1-3 substantially suppressed clonogenic potential in A549 (*p* < 0.05; Fig. 6C), while L1R1-6 did so in H1975 (*p* < 0.05; Fig. 6D).

### AChE inhibitors can induce cell apoptosis and cause cell cycle arrest

**Fig. 7:**
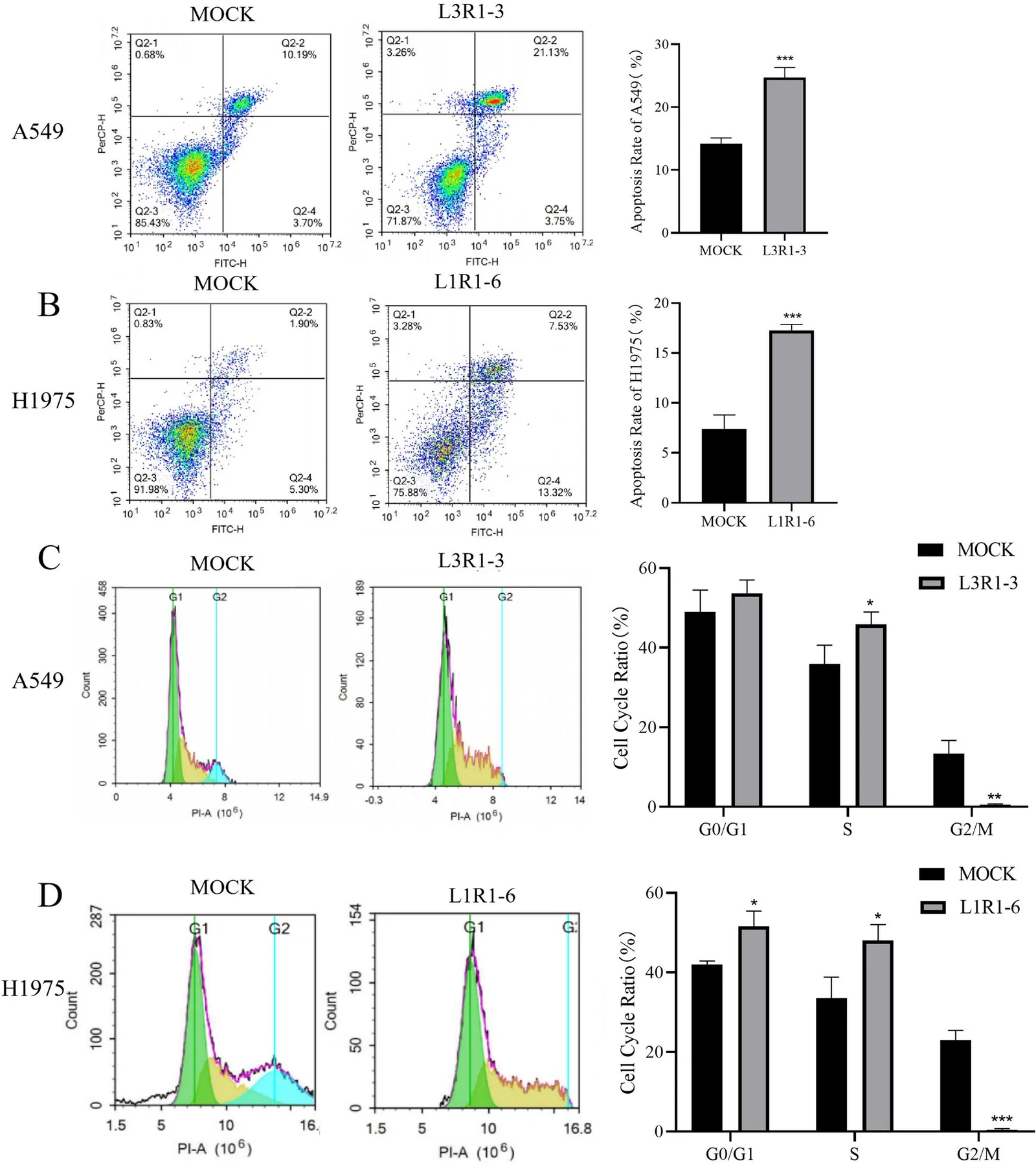
Acetylcholinesterase (AChE) inhibitors induce cell apoptosis and block cell cycle. **A** Apoptosis rate of A549 cells co-cultured with L3R1-3. **B** Apoptosis rate of H1975 cells co-cultured with L1R1-6. **C** Cell cycle distribution of A549 cells co-cultured with L3R1-3. **D** Cell cycle distribution of H1975 cells co-cultured with L1R1-6

At IC_50_ concentrations, L3R1-3 and L1R1-6 significantly induced apoptosis in A549 and H1975 cells, respectively (*p* < 0.05; Fig. 7A and B). Additionally, flow cytometry revealed S phase arrest in A549 (Fig. 7C) and G0/G1 and S phase arrest in H1975 (Fig. 7D).

### AChE inhibitors suppress the growth of tumor cells in mice

**Fig. 8:**
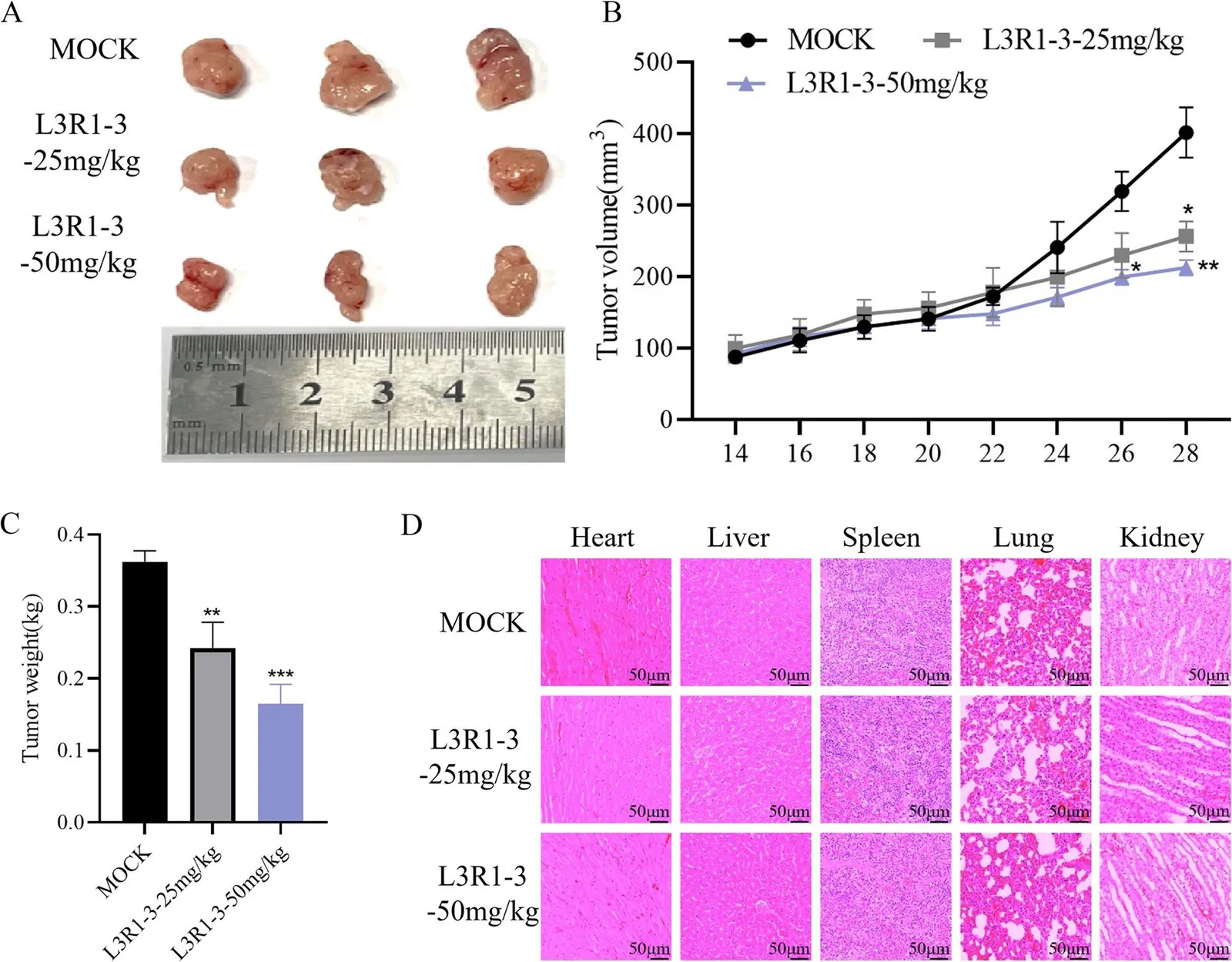
L3R1-3 inhibits the development of lung adenocarcinoma tumors in vivo. **A** Statistical analysis of tumor volume changes in nude mice after injection of L3R1-3. **B** Comparison of tumor volume size after injection of L3R1-3 into nude mice. **C** Comparison of tumor weight after injection of L3R1-3 into nude mice. **D** Histological examination of organ tissues in nude mice stained with hematoxylin and eosin (H&E)

Intraperitoneal injection of the AChE inhibitor L3R1-3 into a nude mouse model of transplanted tumors further confirmed its inhibitory effect on the growth of A549 tumor cells. After an intraperitoneal injection of L3R1-3, the volume of the transplanted tumors in mice became significantly smaller than that in the control group (*p* < 0.05; Fig. 8A and B); the tumor weight was also significantly lower than that in the control group (*p* < 0.05; Fig. 8C). Notably, the 50 mg/kg group showed a more significant reduction in tumor volume and weight.

### AChE inhibitors do not damage normal organs of transplanted tumor mice

Tissue sections were prepared by dissecting the heart, liver, spleen, lungs, and kidneys of the transplanted tumor mice. H&E staining of these tissue sections confirmed that L3R1-3 did not cause any pathological damage to the organs of the transplanted tumor mice (Fig. 8D).

## Discussion

Although many treatment strategies are available for lung cancer in clinical practice, including chemotherapy, targeted therapy, immune checkpoint inhibitors, and antibody–drug conjugate, its high incidence and mortality rates, along with the fact that existing treatments still fall short of meeting clinical needs, make it a key research topic.

AChE has been widely studied as a therapeutic target in neurological diseases, such as Alzheimer’s disease and myasthenia gravis. However, an increasing number of studies have reported on the correlation between AChE and the occurrence and development of various tumors [14]. In some malignant tumors, ACh acts as an autocrine or a paracrine growth factor, promoting tumor progression [18]. In others, it may show abnormal expression or may undergo gene amplification or mutation. Both these situations affect the malignant biological behavior of the tumors. In view of this, research scholars have begun to analyze the relationship between AChE and tumors and evaluate whether it can be used as a target for tumor therapy.

Cancer cells typically exhibit reduced AChE activity to maintain their elevated ACh levels, thereby enhancing their proliferation and invasion potential [19]. Some studies support the above viewpoint and have reported significantly lower AChE activity in colorectal tumors than that in adjacent normal tissues [20]. Wang et al. demonstrated a decrease in AChE expression in cancer cells. They also developed a transferrin-modified liposome delivery system to target AChE expression, which resulted in promising therapeutic effects [16]. However, other studies have reported contrasting results, that is, AChE is highly expressed in some tumors compared to that in normal tissues, such as ovarian cancer [21].

Further research and analysis have shown that the expression or functional activity of cholinergic proteins were upregulated or downregulated in tumors compared to that in paired normal tissues, including cholinergic acetyltransferase (ChAT), vesicular acetylcholine transporter (VAChT), nicotinic acetylcholine receptors (nAChRs), muscarinic acetylcholine receptors (mAChRs), cholinergic transporter like protein 1–5 (CTLP1-5), acetylcholinesterase (AChE), and butyrylcholinesterase (BChE) [14, 22–24], further supporting the role of the cholinergic system in cancers.

This study analyzed the TIMER2.0 database and found that AChE is significantly overexpressed in lung adenocarcinoma. This result is consistent with that reported by An et al., who, using the CPTAC database, reported significantly increased expression of AChE in lung adenocarcinoma, head and neck squamous cell carcinoma, glioblastoma multiforme, and lung squamous cell carcinoma tumors compared to that in the corresponding normal tissues [15].

In view of this, we here designed two AChE inhibitors, small-molecule L3R1-3 and L1R1-6, to demonstrate the role of AChE in lung adenocarcinoma and whether inhibitors can alter the tumor biological behavior. We validated the structures of L3R1-3 and L1R1-6 through NMR, HRMS, and molecular docking analyses. Functional tests showed that these compounds significantly inhibited the proliferation and colony formation of lung adenocarcinoma A549 and H1975 cells and induced their apoptosis. L3R1-3 can induce the S phase arrest in A549 cells, while L1R1-6 can induce the G0/G1 and S phase arrest in H1975 cells. We further validated the above-mentioned inhibitory effect on tumors in a mouse transplant tumor model.

Coincidentally, according to the latest research by Li et al., targeting the cholinergic system can inhibit the brain metastasis of lung adenocarcinoma. This phenomenon can be employed to develop a new strategy for treating lung adenocarcinoma brain metastasis. They demonstrated that the activation of cholinergic metabolism is crucial for lung adenocarcinoma tumors prone to brain metastasis, which is significantly associated with poorer survival outcomes. Subsequent mechanistic studies have shown that AChE promotes brain metastasis through a dual pro-metastatic mechanism: immunosuppression and disruption of the blood–brain barrier. In addition, choline acetyltransferase enhances tumor invasiveness by stimulating proliferation and migration of cancer cells. Hence, targeting ACh could be a useful therapeutic strategy [17]. This study further confirms the impact of ACh on the biological behavior of lung adenocarcinoma, which is highly consistent with our viewpoint. AChE inhibitors have been studied in cell lines such as HepG2 and melanoma, which also confirms that targeting ACh may become a potential therapeutic strategy [25–26].

Despite the robust anti-tumor phenotypes observed, the detailed molecular mechanism by which our AChE inhibitors affect lung adenocarcinoma remains incompletely defined. Based on the pharmacological properties of AChE inhibition and cholinergic signaling in epithelial cancers, we propose a testable mechanistic framework centered on PI3K/AKT and MAPK/ERK pathway attenuation, coupled with BCL-2 family-regulated apoptosis.

Acetylcholine (ACh) signals through nicotinic and muscarinic receptors (nAChRs and mAChRs, respectively) to promote proliferation and survival via PI3K/AKT and MAPK/ERK cascades. AChE inhibition is expected to elevate ACh, paradoxically inducing receptor desensitization and dampening downstream AKT and ERK phosphorylation. This dual suppression reduces mTORC1-mediated survival signaling, relieves FOXO-mediated cell cycle inhibition, and impairs cyclin D1/E2F activity, providing a plausible explanation for proliferation arrest and S-phase/G₀/G₁ blockade that we observed. Concurrently, AChE-inhibitor-induced receptor desensitization may reverse the BCL-2/BAX balance, tipping the rheostat toward apoptosis, consistent with the significant Annexin V positivity detected in our assays. These interconnected pathways form a coherent mechanistic scaffold for the anti-tumor activities of L1R1-6 and L3R1-3.

However, this model remains inferential and requires systematic validation through phospho-specific analyses, receptor subtype-specific agonists/antagonists, and gene-silencing approaches. The non-classical scaffolding functions of AChE and potential compensation by butyrylcholinesterase (BChE) also warrant investigation. Furthermore, whether AChE inhibition reshapes the tumor immune microenvironment to contribute to tumor suppression remains an open question. Importantly, independent replication in other lung adenocarcinoma models, including PDX and genetically engineered mouse models, is essential to establish AChE as a firm therapeutic target. Future studies should also explore combination strategies with chemotherapy or immunotherapy, given the emerging role of cholinergic signaling in immune modulation. Comprehensive pharmacokinetic and toxicity profiling will be necessary before clinical translation of AChE.

## Conclusion

This study conducted a comprehensive bioinformatics and experimental investigation of AChE in lung adenocarcinoma. We demonstrated that AChE is significantly overexpressed in lung adenocarcinoma tissues and cell lines. These findings highlight the potential of these novel AChE inhibitors in the treatment of lung adenocarcinoma, including their ability to effectively inhibit cell proliferation, induce apoptosis, prevent cell cycle progression, and reduce the colony-forming ability. Using a mouse transplant tumor model, this study also confirmed that AChE inhibitors can inhibit cell proliferation, without causing any pathological damage to normal organs.

Our research methodology spans bioinformatics analysis, small-molecule compound development, biological behavior research, establishment of mouse transplant tumor models, and empirical evidence, providing strong evidence for the therapeutic value of AChE inhibitors in lung adenocarcinoma. Overall, our research findings emphasize that AChE is a potential therapeutic target for treating lung adenocarcinoma and suggest that AChE inhibitors may be a promising new approach for treating this invasive tumor. Further in vivo and mechanistic studies are needed for validating the clinical applicability of our proposed AChE-based therapies and to elucidate the precise molecular pathways involved.

## Supplementary Information

Supplementary Material 1.

## Data Availability

All data supporting the findings of this study are available within the paper and its Supplementary Information.

## Abbreviations

AChE: Acetylcholinesterase
NSCLC: Non-small cell lung cancer
EGFR: Epidermal growth factor receptor
ALK: Anaplastic lymphoma kinase
ORR: Objective response rate
DoR: Duration of efficacy
Ach: Acetylcholine
AChE-T: Tail type AChE isoforms
AChE-H: Dimer H subtype AChE
AChE-R: Reading type AChE
H&E: Hematoxylin and eosin
ChAT: Cholinergic acetyltransferase
VAChT: Vesicular acetylcholine transporter
NAChRs: Nicotinic acetylcholine receptors
MAChRs: Muscarinic acetylcholine receptors
CTLP1-5: Cholinergic transporter like protein 1–5
BChE: Butyrylcholinesterase
